# Interfacing High-Throughput Electrosynthesis and Mass Spectrometric Analysis of Azines

**DOI:** 10.1021/acs.analchem.4c01110

**Published:** 2024-05-08

**Authors:** Krista M. Kulesa, Erin A. Hirtzel, Vinh T. Nguyen, Dallas P. Freitas, Madison E. Edwards, Xin Yan, Lane A. Baker

**Affiliations:** † Department of Chemistry, Indiana University, Bloomington, Indiana 47405, United States; ‡ Department of Chemistry, Texas A&M University, College Station, Texas 77843, United States

## Abstract

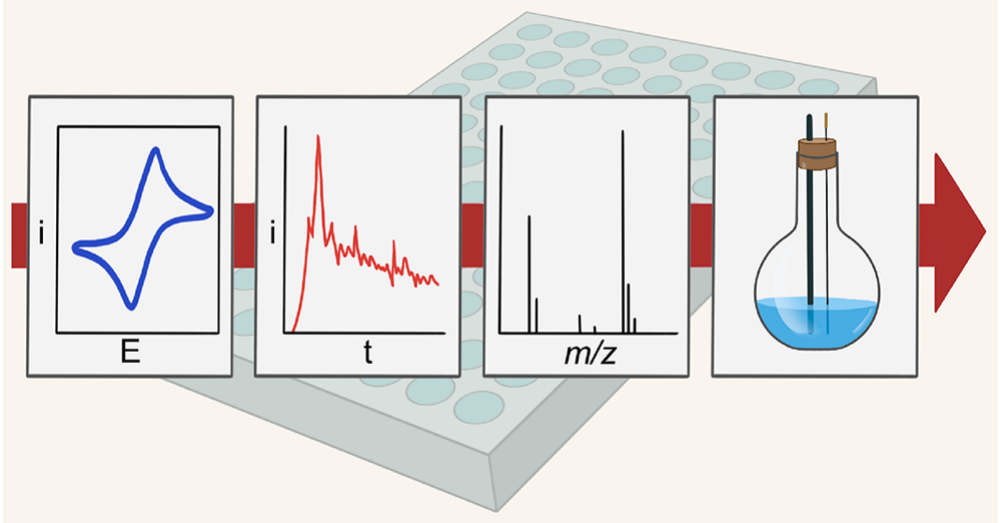

Combinatorial electrochemistry has great promise for accelerated reaction screening, organic synthesis, and catalysis. Recently, we described a new high-throughput electrochemistry platform, colloquially named “Legion”. Legion fits the footprint of a 96-well microtiter plate with simultaneous individual control over all 96 electrochemical cells. Here, we demonstrate the versatility of Legion when coupled with high-throughput mass spectrometry (MS) for electrosynthetic product screening and quantitation. Electrosynthesis of benzophenone azine was selected as a model reaction and was arrayed and optimized using a combination of Legion and nanoelectrospray ionization MS. The combination of high-throughput synthesis with Legion and analysis via MS proves a compelling strategy for accelerating reaction discovery and optimization in electro-organic synthesis.

High-throughput screening has made critical contributions to accelerated hypothesis testing, discovery, and data handling in numerous research fields, including biochemistry,^1−4^ drug discovery,^5,6^ synthesis and reactivity,^7−11^ catalysis,^12−15^ and machine learning.^16−19^ Although significant progress has been made, wide-scale adoption of high-throughput electrochemistry (HTE)^20−23^ has consistently lagged in development compared to other analytical methods, such as electron microscopy and mass spectrometry (MS). Given the sensitivity and information density of electrochemical techniques, high-throughput electrochemical approaches, particularly when coupled to complementary forms of high-throughput analysis, would have a significant impact on the fields of catalysis and electrosynthesis.

We have recently described a platform for high-throughput electrochemistry, called “Legion,” which is capable of simultaneous, individual, arrayed electrochemical measurements across all electrochemical cells.^24^ Moreover, to facilitate complementary analysis tools such as MS, the footprint of Legion is modeled after a commercial 96-well microtiter plate.

HTE has significant promise in the field of electrosynthesis. Through a combination of arrayed electrochemistry and nanoelectrospray ionization (nESI) MS methods (Figure 1), we study the oxidative N–N homocoupling reaction of benzophenone imine to form hydrazine in the protected form of benzophenone azine (Figure 2a). Originally studied by Stahl et al.,^25^ the reaction was ideal to demonstrate interfacing Legion with MS: all reagents are water- and air-tolerant, both the starting material and product have high ionization efficiencies, and the selected supporting electrolyte minimizes salt suppression in postelectrolysis mass spectra. These features collectively enable MS analysis to complement HTE. High-throughput mass spectrometric methods provide rapid access and molecular specificity to chemical reactivity. Recognized as a powerful and versatile tool for high-throughput reaction screening,^26−31^ MS facilitates the analysis of a wide range of molecules. Electrochemistry has previously been coupled with MS,^32,33^ with significant success, particularly in serial array approaches demonstrated by Mayrhofr and co-workers.^34−39^ As demonstrated here, standard MS seamlessly interfaces with the standard footprint of the well plate of Legion for the characterization of conversion ratios (CRs) in electrochemical reactions. Legion-MS experiments expedite parameter screening to optimize this reaction, allowing for a direct scale-up.

**Figure 1:**
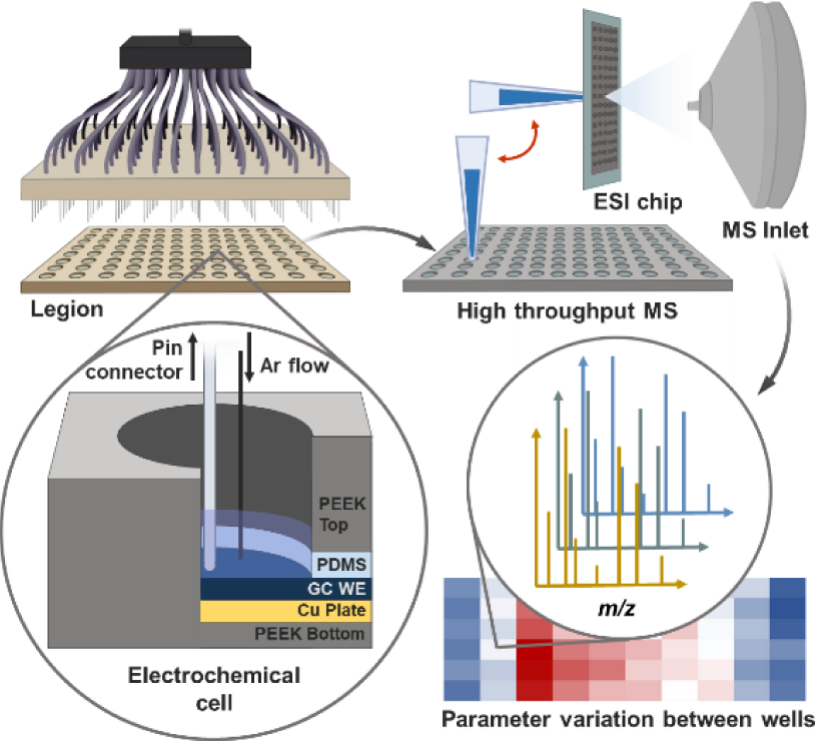
Workflow of Legion-MS: high-throughput electrochemistry experiments interfaced with automated, high-throughput mass spectrometry. Mass spectra collected across the arrayed plate produce a heatmap of relative conversion from imine to azine.

**Figure 2:**
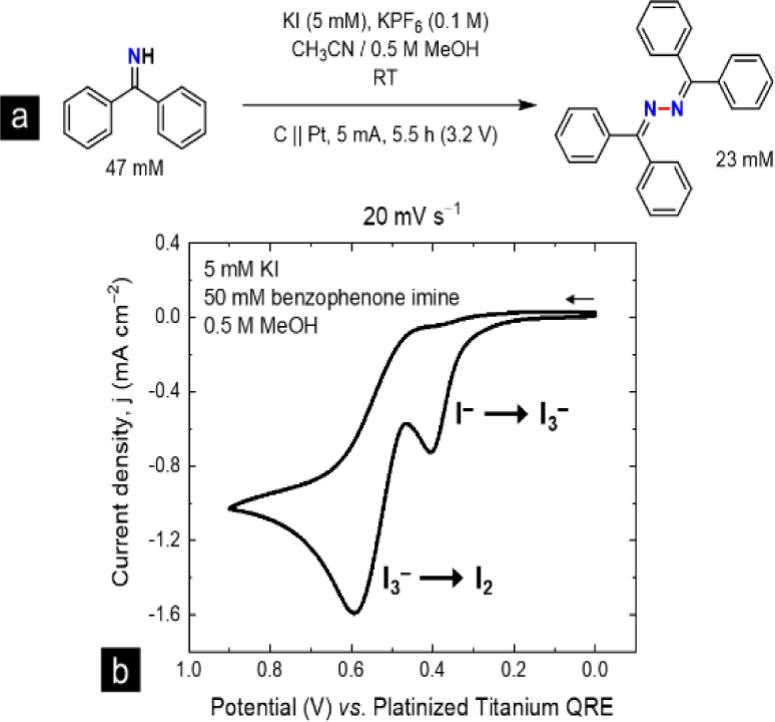
(a) Electrosynthesis of benzophenone azine from two equivalents of benzophenone imine, optimized by combinatorial methods. (b) Cyclic voltammogram of the reaction mixture: 5 mM potassium iodide, 50 mM benzophenone imine starting material, and 500 mM methanol in 0.1 M KPF_6_ supporting electrolyte in acetonitrile. At a glassy carbon working electrode, the active catalyst I_2_ is generated from two irreversible 1 e^–^ oxidations (ferrocene/ferrocenium = +0.042 V vs platinized titanium).

## Experimental Section

### Legion HTE

Instrument design of Legion is previously reported by Gerroll et al.^24^ Each well is an individual electrochemical cell with a two-electrode configuration. The working electrode is a glassy carbon plate or graphite plate (redox.me) with an exposed electroactive surface area of 38.5 mm^2^ per well. Each well contains 300 μL of analyte solution. 96 quasi-reference counter electrodes (QRCEs) extend into solution and connect to the field-programmable gate array (FPGA) control board. These measurements utilize 1 mm diameter platinized titanium wire QRCEs (Ti-shop, William Gregor Limited), with electroactive surface areas of approximately 35 mm^2^. Preparative scale experiments utilized a graphite rod (6.8 mm diameter; Alfa Aesar, 99%) working electrode; platinum coil (1 mm diameter, Alfa Aesar) counter electrode; and platinized titanium or Ag wire quasi-reference electrode. More experimental details can be found in the Supporting Information.

### HT-MS Analysis

Product arrays were analyzed by high-throughput nESI-MS via Advion TriVersa NanoMate (Ithaca, NY), and the results were confirmed with glass capillary nESI-MS (see details in Supporting Information). To adjust analytes to appropriate concentrations for MS analysis and reduce potential ion suppression effects caused by the presence of an electrolyte, samples were diluted by a factor of 1000 with acetonitrile prior to analysis. The TriVersa NanoMate is a chip-based nESI platform, enabling the automated, high-throughput MS infusion analysis of reaction arrays. This platform ensures precise control over the sampling volume through programmed pipetting of the reaction mixture and interfaces directly with mass analysis, which was executed on a Thermo Fisher Scientific LTQ XL.

## Results and Discussion

### High-Throughput Reaction Parameter Evaluation

Cyclic voltammetry of this reaction was initially performed with a CHI 660C commercial potentiostat/galvanostat, using a three-electrode configuration: glassy carbon (3 mm diameter) working electrode; platinum wire (1 mm diameter) counter electrode; and platinized titanium wire (1 mm diameter) quasi-reference electrode. Measurements were made in acetonitrile with 0.1 M KPF_6_ as supporting electrolyte, against a platinized titanium (Pt–Ti) quasi-reference electrode with ferrocene as an internal standard. At 20 mV s^–1^, two electron transfer events are observed as irreversible voltammetry oxidations, or processes, in the presence of benzophenone imine, potassium iodide, and methanol. In good agreement with the literature, the first electron transfer occurs ca. 0.47 V vs Pt–Ti. This irreversible process is attributed to the electrochemical oxidation of I^–^ to I_3_^–^ (I^–^/I_3_^–^). A second irreversible oxidation occurs at 0.59 V vs Pt–Ti, which is likely I_3_^–^/I_2_ (Figure 2b).^40,41^ At faster scan rates, I^–^/I_3_^–^ becomes more reversible, with a reduction process corresponding to I_3_^–^/I^–^ appearing at 0.10 V vs Pt–Ti on the return sweep. At 100 mV s^–1^, reduction of I_3_^–^ back to I^–^ occurs at the expense of I_2_ generation. This suggests a chemical rate-determining step for iodine-catalyzed benzophenone imine oxidation. Since I_2_ is the active catalyst, sufficient concentrations of I_2_ must be generated at the electrode surface to promote benzophenone azine formation. Faster scan rates do not allow enough time for oxidation of I_3_^–^ to I_2_.

**Figure 3:**
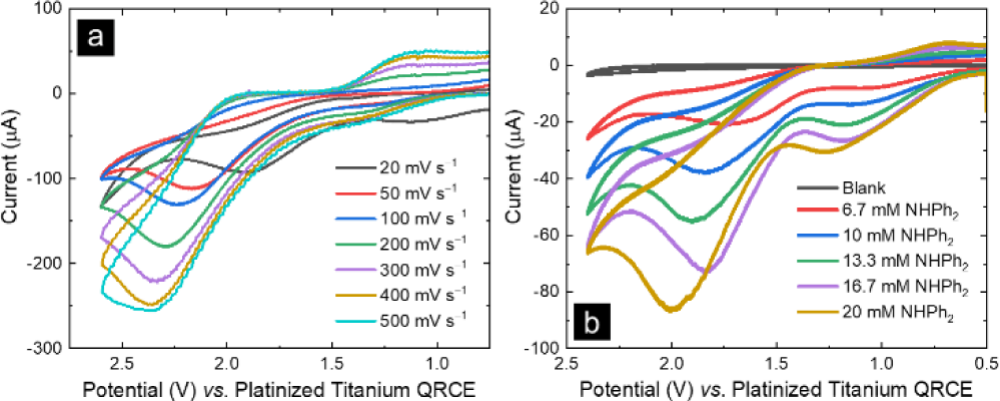
(a) Variable scan rate studies of the reaction mixture 2 mM KI, 2 mM benzophenone imine (NHPh_2_), and 200 mM H_2_O, in 0.1 M KPF_6_ supporting electrolyte in acetonitrile. Voltammograms with Legion show the same trends in reversibility as commercial measurements. (b) Arraying concentrations of benzophenone imine expedites sequential titration experiments and shows a linear dependence of catalytic current on the substrate. Both arrays were measured at a glassy carbon plate working electrode against a platinized titanium wire QRCE. Voltammogram sets (a) and (b) are each plotted as the average measurement across one corresponding set of eight wells.

To validate the performance of Legion compared to commercial instrumentation, cyclic voltammetry was performed in acetonitrile at a glassy carbon plate working electrode and a platinized titanium wire quasi-reference counter electrode. All 96 wells operated in air without deoxygenation. Arrayed variables include the supporting electrolyte, substrate concentration, proton source, and the presence of a catalyst. Analogous to measurements made with commercial instrumentation, two irreversible oxidation processes are observed at 20 mV s^–1^ (1 mV voltage step, 0.5 s time step; Figure 3a). Independent of the supporting electrolyte, KI is necessary to catalyze N–N homocoupling to form the benzophenone azine product. Additionally, arrayed concentrations of benzophenone starting material demonstrate a first-order dependence on the substrate (Figure 3b). This is indicated by a linear increase in catalytic current with increasing concentrations of benzophenone imine (0–20 mM).

Using high-throughput voltammetry as a guide, controlled potential electrolyses were arrayed with Legion and evaluated by MS to optimize the electrosynthesis of benzophenone azine. Electrolysis products were analyzed by chip-based nESI-MS. Controlled potential electrolysis (CPE) was performed at both a glassy carbon plate and a graphite plate working electrode, with a platinized titanium wire quasi-reference counter electrode in each electrochemical cell (Figures S2 and S3). Proton donor, catalyst concentration, substrate concentration, and applied potentials were arrayed across the 96-well plate. Controlled potential electrolyses were run in quiescent solutions to favor a surface-based mechanism. Heatmaps produced from the array were used to guide further optimizations.

In the first stage of discovery, controlled potential electrolysis experiments were performed with Legion and a glassy carbon working electrode. Potentials ranging from 1.8 to 3.3 V were applied over the course of 1 to 5.5 h. All 96 wells also contained a ratio of 2:20:200 mM catalyst/substrate/acid, respectively. At glassy carbon, the reaction was still incomplete after nearly 6 h of electrolysis. Nonetheless, benzophenone azine product was detectable after CPE with applied potentials >2.5 V vs Pt–Ti. More azine product was detected with increasing oxidative potential, up to 2.9 V. At 3.0 V and more positive, the azine yields decreased. During cyclic voltammetry, a third irreversible oxidation process was observed in the presence of KI, benzophenone, imine, and methanol in acetonitrile. The third electron transfer resulted in intractable side products.

An additional variable in the electrosynthesis of benzophenone azine is the source of protons for H_2_ evolution in the counter reaction at the cathode. During H_2_ evolution, the conjugate base diffuses to the working anode to deprotonate the benzophenone imine as an initial step in the catalytic cycle. During high-throughput CPE, the surface area mismatch between the working electrode and quasi-reference counter electrode forces H_2_ evolution to support the electrocatalytic current. We hypothesize this results in a cleaner target reaction and a more alkaline electrolyte, disfavoring the acid-catalyzed hydrolysis of benzophenone azine into hydrazinium. Over the course of CPE, wells containing H_2_O/OH^–^ demonstrated higher yields of the azine product.

**Figure 4:**
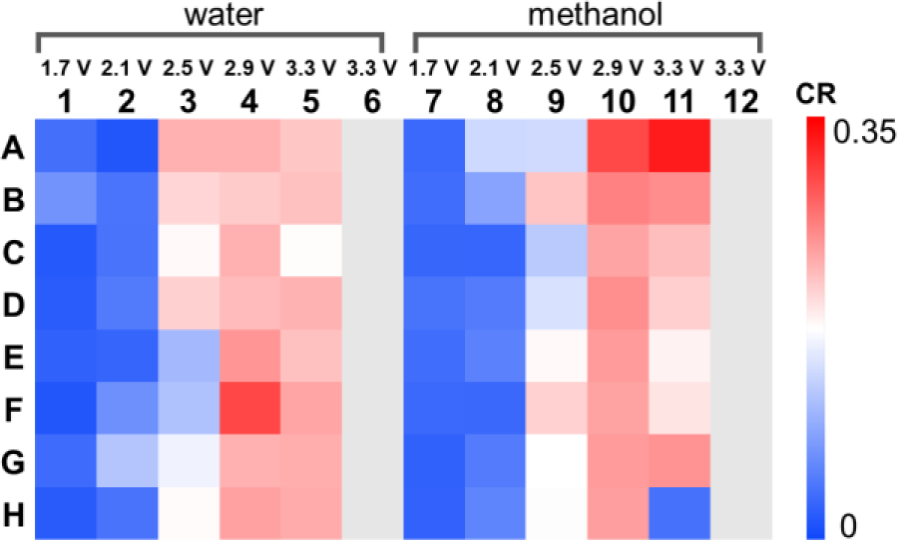
Heatmap for electrosynthesis of benzophenone azine with glassy carbon using different potentials and proton source, analyzed by chip-based nESI-MS. Heatmap was generated using the conversion ratio (CR) calculated with MS ion intensities. Columns 6 and 12 contain only supporting electrolyte and therefore have no CR value.

Heatmap analysis comparing relative conversion of reactant to product confirms the impacts of applied potential and acid source on azine electrosynthesis (Figure 4). Conversion ratio (CR) was calculated using the MS ion intensity of product ions (protonated, sodiated, and potassiated benzophenone azine) over the sum of the product and reactant ion intensities. In the heatmap, blue represents low concentrations of electrosynthesis product, while red represents high concentrations.

Legion experiments were also performed at a graphite plate working electrode. Similar to literature reports, reactivity at graphite substantially improves compared to that on a glassy carbon surface. Proton source and applied potential were arrayed across the well plate. Compared to glassy carbon, optimal azine formation at a graphite working electrode occurs at an applied potential of 2.9 V. Potentials less oxidizing than 2.7 V and more oxidizing than 3.1 V result in lower electrolysis product yields, and additional unidentifiable products by MS. After 3 h, wells containing water as a proton source (OH^–^ as a conjugate base) and with an applied potential of 2.9 V formed the most product. Unlike reported CPE yields, Legion arrays produce up to 44% CR of benzophenone azine.

Despite greater heterogeneities across the graphite plate (Figure S4), azine yields are consistently higher than those at glassy carbon. Moreover, these comparatively high yields are in the presence of water and oxygen, whereas literature precedent conducts electrolyses in rigorously dried solvents and glassware under inert atmospheres. However, after >3 h, wells containing methanol produce more benzophenone azine. This is likely due to coupled water reduction to form H_2_ at the cathode and competing water oxidation at the anode. As water is reduced to H_2_, the electrolyte basifies at the cathode surface and lowers the overpotential for O_2_ evolution. In addition, electrogenerated hydride may react with acetonitrile to form the imine condensation product. To avoid O_2_ evolution and heterocoupling side products during prolonged electrolysis, the MeOH/MeO^–^ pair was selected for scale-up experiments at graphite.

### Bulk Analysis of Optimized Parameters

**Figure 5:**
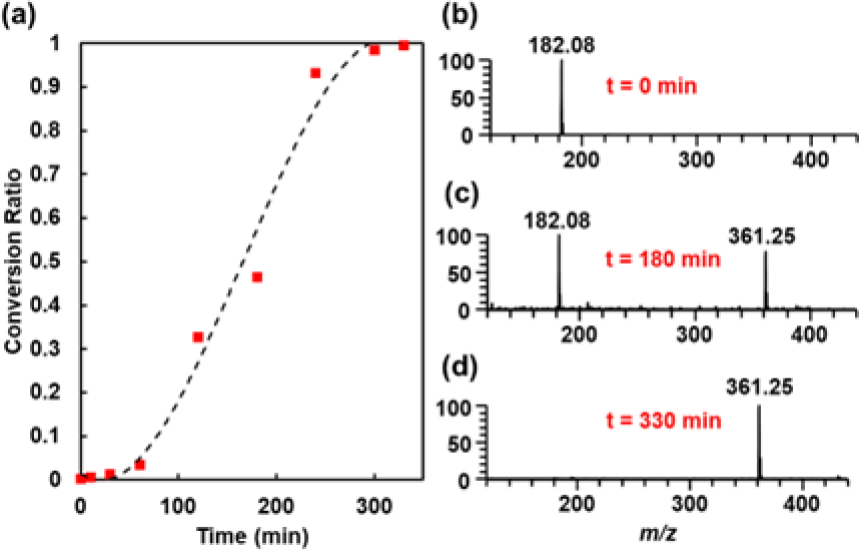
Kinetic study of electrocatalytic benzophenone azine formation in bulk over time via nESI-MS. (a) Plot of the CR of azine product with 1000× dilution in acetonitrile. Mass spectra of the reaction mixture sampled at (b) 0, (c) 180, and (d) 330 min.

To translate Legion electrochemistry to the preparative scale, benzophenone azine electrosynthesis was performed using a DC power source. Constant current electrolysis was carried out using 0.1 M KPF_6_ supporting electrolyte in acetonitrile, with a two-electrode configuration: a graphite rod anode and platinum coil cathode. Under an ambient air atmosphere, the working electrode was held at a 5 mA current in an undivided cell. Aliquots of the electrolysis solution were taken at different time points, and calculated CRs at these intervals were used to assess product formation (Figure 5). The final MS analysis at 330 min yielded a CR of 0.996, and the obtained MS spectrum was comparable to that of a commercially available standard at the same concentration assuming 100% conversion (Figure S6).

At the onset of electrolysis, the solution changed color rapidly from colorless to bright yellow and then back to colorless, due to the generation and rapid consumption of I_2_ in acetonitrile. Over the course of electrolysis, the solution turned yellow once more. The solution eventually darkened to orange with a sparingly soluble white precipitate, which was the benzophenone azine product. After electrolysis, the product was collected, extracted into benzene, purified by flash chromatography, and dried *in vacuo* for analysis. By both ^1^H NMR and MS, the constant current electrolysis product was comparable in purity to that of the commercially obtained compound (Figure S7). The reaction was found to be water- and oxygen-tolerant and to proceed with quantitative yields. Rapid hypothesis testing via combinatorial electrochemistry and automated MS was critical to scaling and optimizing this reaction as well as understanding its adaptability.

In total, electrochemical arrays interfaced with MS provide an intriguing tool for rapid reaction development and product identification. As a proof of concept, electrosynthesis of the compound benzophenone azine was studied through Legion-MS. Hypothesis testing toward reaction optimization surpassing literature reports is facilitated greatly with the unique simultaneous, individual experimental capabilities of Legion’s design. High-throughput experiments with Legion lead to significant conversion of benzophenone azine by controlled potential electrolysis and quantitative preparative-scale yields by constant current electrolysis. Insights from both types of experiments suggest a surface-sensitive rate-determining step, which informs pure, scalable benzophenone azine analogs, as well as broader electrosynthetic reactions.

## Supporting Information Available

Details on experimental configurations, controlled potential electrolysis data, MS, and NMR spectroscopy (PDF)
