# Supplementary material for: Interfacing High-Throughput Electrosynthesis and Mass Spectrometric Analysis of Azines
Source: Anal Chem. 2024 May 8;96(21):8249–53. doi: 10.1021/acs.analchem.4c01110 (PMC11140680; doi:10.1021/acs.analchem.4c01110)
Supplement: Supplementary file 1 — ac4c01110_si_001.pdf [file ac4c01110_si_001.pdf]

Supporting Information

Interfacing High-Throughput Electrosynthesis and Mass Spectrometric Analysis of Azines

Krista M. Kulesa,[†,‡,#] Erin A. Hirtzel,[‡,#] Vinh T. Nguyen,[‡] Dallas P. Freitas,[‡] Madison E. Edwards,[‡] Xin Yan,[‡*] and Lane A. Baker[‡*]

[†] Department of Chemistry, Indiana University, Bloomington, Indiana 47405
[‡] Department of Chemistry, Texas A&M University, College Station, Texas 77843

*Corresponding author emails: xin.yan@chem.tamu.edu ; lane.baker@chem.tamu.edu

[#] K.M.K. and E.A.H. contributed equally to this paper.

Table of Contents

Experimental Methods **S-2**

High-throughput electrochemistry **S-2**

Electrodes **S-3**

Commercial voltammetry **S-3**

Constant current electrolysis (CCE) **S-4**

Mass spectrometry (MS) **S-4**

Figure S1 – Distribution in current outputs (μA) across the 96-well plate at varied scan rates **S-6**

Figure S2 – Arrayed controlled potential electrolysis (CPE) for approximately 5.5 hours at a glassy carbon working electrode surface **S-7**

Figure S3 – Arrayed controlled potential electrolysis (CPE) for approximately 2.75 hours at a graphite working electrode surface **S-8**

Figure S4 – Heatmap for electrosynthesis of benzophenone azine with graphite **S-9**

Figure S5 – Distribution of conversion ratios across the 96-well plate **S-10**

Figure S6 – Comparison by mass spectrometry of (**a**) electrosynthesized benzophenone azine, and (**b**) commercial benzophenone azine **S-11**

Figure S7 – ^{1}H-NMR in CD_3CN of (**a**) commercial benzophenone azine (*teal*), and (**b**) electrosynthesized benzophenone azine (*dark red*) **S-12**

References **S-13**

Experimental Methods

High-throughput electrochemistry

Instrument design of Legion is previously reported by Gerroll *et al.*[1] Legion is custom-built, modeled around a 96-well microtiter plate to facilitate secondary analysis. Each well is an individual electrochemical cell, arranged in a 12 x 8 format. The assembly is comprised of three well plates machined from polyether ether ketone (PEEK). The bottommost plate holds a copper sheet which forms a conductive contact with the working electrode. The working electrode is a glassy carbon plate (110 mm x 73 mm x 3 mm) or graphite plate (107 x 71 x 3.5 mm). The working electrode plate also forms the bottom of each well. The exposed electroactive surface area is 7 mm in diameter (38.5 mm^2). Each well has a maximum capacity of 500 μL, and minimum working capacity of 200 μL. All experiments are carried out on 300 μL of analyte solution. Wells are divided from one another by a polydimethylsiloxane (PDMS) gasket and a second PEEK plate. The height of the second PEEK plate defines the depth of each well. The conductive base plate, working electrode, gasket, and PEEK plates are compressed in place by corner and lateral screws.

A third, topmost PEEK plate houses 96 quasi-reference counter electrodes (QRCEs), which interface directly with the field-programmable gate array (FPGA). These measurements utilize 1 mm diameter platinized titanium wire QRCEs, with electroactive surface areas of approximately 35 mm^2. Each QRCE interfaces with the instrument *via* pin connectors which lead to customized circuitry and electronics for the potentiostat. Eight QRCEs connect to an 8-channel FPGA board to form one channel; the potentiostat consists of twelve 8-channel FPGA boards. Current is measured through the QRCE across a sensing resistor in the counter electrode channel. Instrument software is custom designed in Labview. Legion performs sweep, step, and electrolysis measurements with an operating voltage range of $\pm$4 V and current range of $\pm$ 250 μA.

Electrodes

A glassy carbon plate (110 mm x 73 mm x 3 mm; redox.me) or graphite plate (107 x 71 x 3.5 mm; vendor) functions as the working electrode and floor of each well. The glassy carbon plate is polished on a MasterTex Buehler polishing pad, first in 0.3 µm grit alumina slurry, followed by a 0.05 µm grit alumina slurry. In between each grit, the plate is sonicated in an activated charcoal-ethanol suspension. Finally, the plate is polished on a bare wet polishing pad, then sonicated in the anticipated experimental solvent. During glassy carbon-based experiments, all cells are enabled.

For measurements at a graphite working electrode, the graphite plate is sonicated in ethanol, wiped clean with a Kimwipe, and sanded with 2500 Grit wet-to-dry sandpaper. The graphite is sonicated again, polished to a mirror-finish with glossy paper, rinsed and air-dried. During graphite-based experiments, perimeter cells are disabled.

All QRCEs are 1 mm diameter metal wires cut to 25 mm length. Platinized titanium wires (Ti-Shop, William Gregor Limited) are sanded with 2100 Grit wet-to-dry sandpaper, then rinsed with ultrapure water and ethanol before use.

Commercial voltammetry

Cyclic voltammetry of this reaction was initially measured using a CHI 660C commercial potentiostat/galvanostat. Cyclic voltammetry was performed in a single-compartment dram cell using a 3-electrode configuration: glassy carbon (3 mm diameter) working electrode; platinum wire counter electrode (1 mm diameter); platinized titanium quasi-reference electrode (1 mm diameter). All measurements were made in HPLC-grade acetonitrile (Fisher Chemical, 99.9%; used as purchased) with 0.1 M KPF_6 supporting electrolyte and ferrocene as an internal standard, without deoxygenation.

Constant current electrolysis (CCE)

Scale-up constant current electrolysis (CCE) experiments were carried out in a 25-mL 3-necked round bottom flask glass cell using a 2-electrode configuration: graphite rod working anode (6.7 mm diameter); platinum coil counter cathode (1 mm diameter). An Agilent E3612A DC power supply was used to hold a 5 mA current during electrolysis.

CCE products were characterized by NMR and MS. ^{1}H-NMR measurements were conducted using a Bruker Avance Neo 400 Hz and 500 Hz NMR, and analyzed by MestreNova. All NMR measurements were made in CD_3CN at room temperature, with mesitylene as an internal standard.

Mass spectrometry (MS)

For MS analysis, nESI emitters were made from borosilicate glass tubing (World Precision Instruments, Sarasota, FL) on a P-100 micropipette puller (Sutter Instrument Company, Novato, CA) using the following parameters: heat 530, pull 0, velocity 15, time 250, and pressure 500. Chip-based direct infusion by NanoMate was achieved by up taking 5.0 μL of sample and delivering the sample for 30 seconds with a gas pressure of 0.40 psi. For both analyses, a voltage of 1.70 kV was used, and samples were diluted 1000x by acetonitrile. Product analysis was performed on a Thermo Fisher Scientific LTQ XL (San Jose, CA). The following MS parameters were set for data acquisition: positive mode, capillary temperature 275 °C, capillary voltage 11 V, tube lens voltage 50 V, and m/z 100-500.

Product arrays were analyzed by nESI-MS *via* Advion TriVersa NanoMate (Ithaca, NY), and the results were confirmed with glass capillary nESI-MS. The TriVersa NanoMate is a chip-based nESI platform, enabling automated, high-throughput MS infusion analysis of reaction

arrays. This platform ensures precise control over the sampling volume through programmed pipetting of the reaction mixture and interfaces seamlessly with mass analysis, which was executed on a Thermo Fisher Scientific LTQ XL (based in San Jose, CA). Mass spectra were processed in Xcalibur Qual Browser (Thermo Fisher Scientific). Heatmaps were generated in Microsoft Excel.

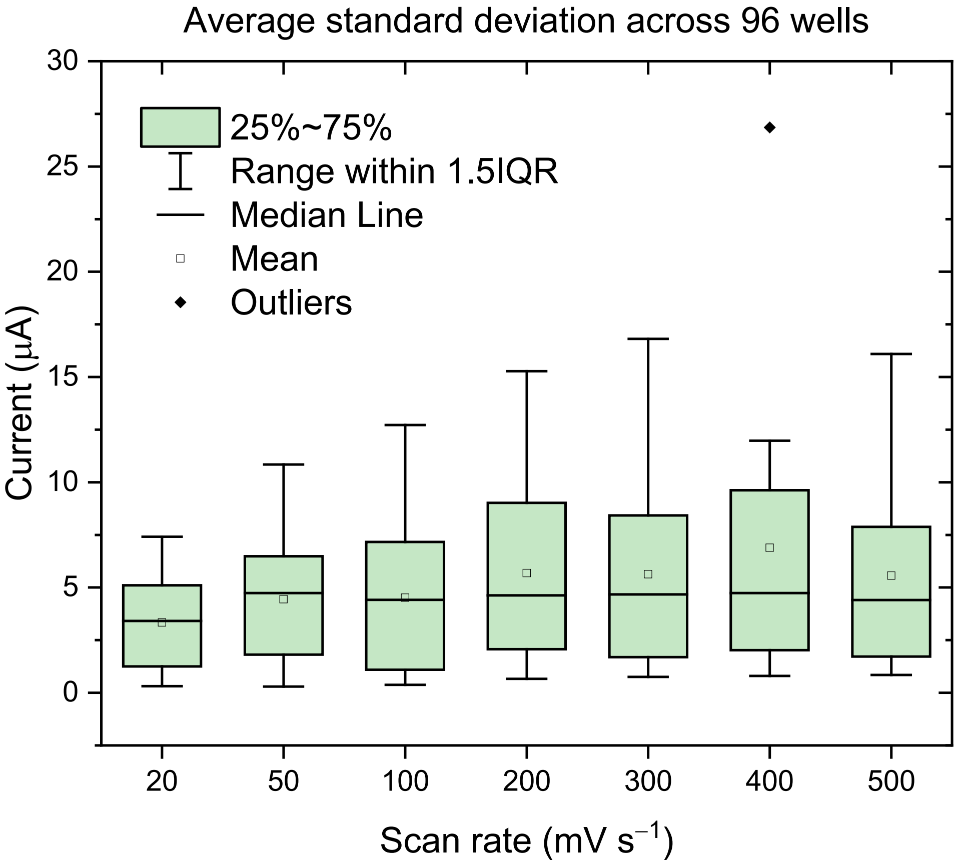

Figure S1. Distribution in current outputs (μA) across the 96-well plate at varied scan rates. Each bar represents the average standard deviation across all 12 channels at one scan rate, calculated from the average standard deviations between 8 wells in one channel.

Arrays contain a 2-electrode configuration: glassy carbon plate working electrode and platinized titanium (Pt-Ti) wire quasi-reference counter electrode. Cyclic voltammetry is conducted in 300 μL of 0.1 M KPF_6 supporting electrode in acetonitrile, with 2 mM KI, 20 mM benzophenone imine, and 200 mM of HA / A^- (HA = methanol or water).

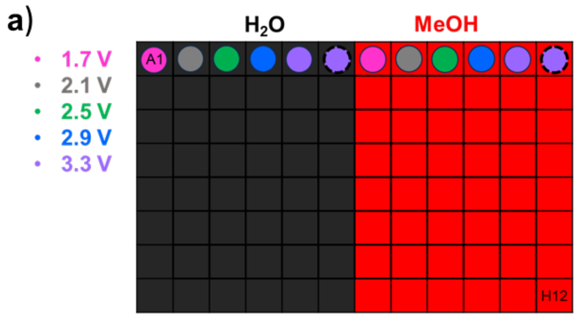

b) Charge consumption, Q (Coulombs)

Voltage	Water	Methanol
1.7 V	0.0906 C	0.0174 C
2.1 V	0.1309 C	0.1582 C
2.5 V	0.2305 C	0.2848 C
2.9 V	0.4638 C	0.4949 C
3.3 V	0.4185 C	0.4298 C

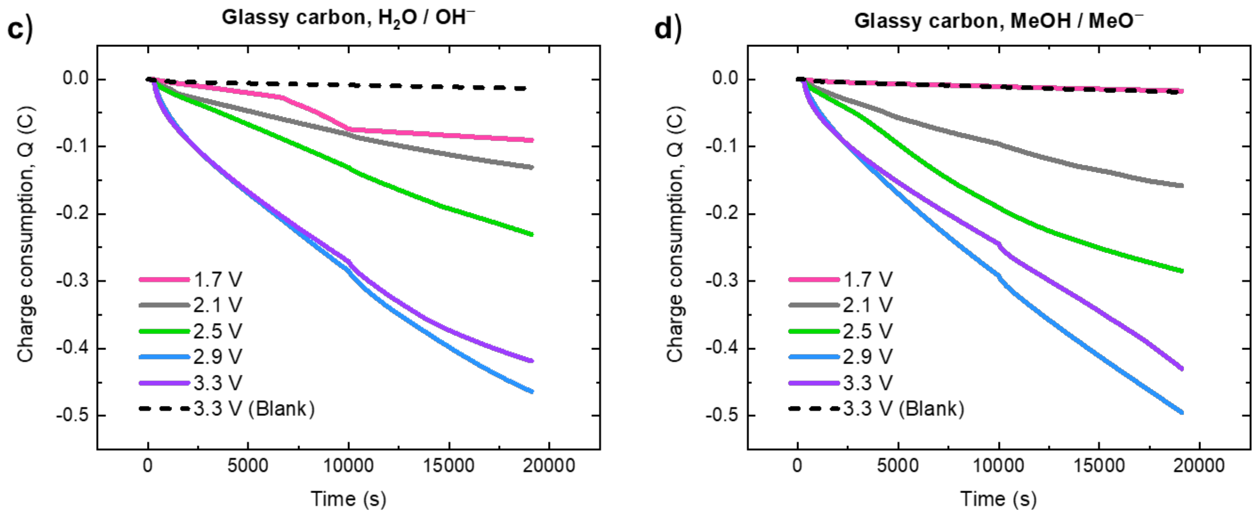

Figure S2. Arrayed controlled potential electrolysis (CPE) for approximately 5.5 hours at a glassy carbon working electrode surface. Arrays contain a 2-electrode configuration: glassy carbon plate working electrode and platinized titanium (Pt-Ti) wire quasi-reference counter electrode. Electrolyses are conducted in 300 μL of 0.1 M KPF_6 supporting electrode in acetonitrile, with 2 mM KI, 20 mM benzophenone imine, and 200 mM of HA / A^{-}.

Arrays consist of (a) water (*black*) and methanol (*red*) as acids and conjugated bases, as well as varying applied potentials. (b) Charge consumption over the course of 6 hours of electrolysis indicates that potentials more positive than 2.9 V *vs*. Pt-Ti lead to decreased product yields. Charge *vs*. time plots in the presence of (c) 200 mM water, and (d) 200 mM methanol. Each charge *vs*. time plot and charge consumption value represents the average measurement across one corresponding set of eight wells.

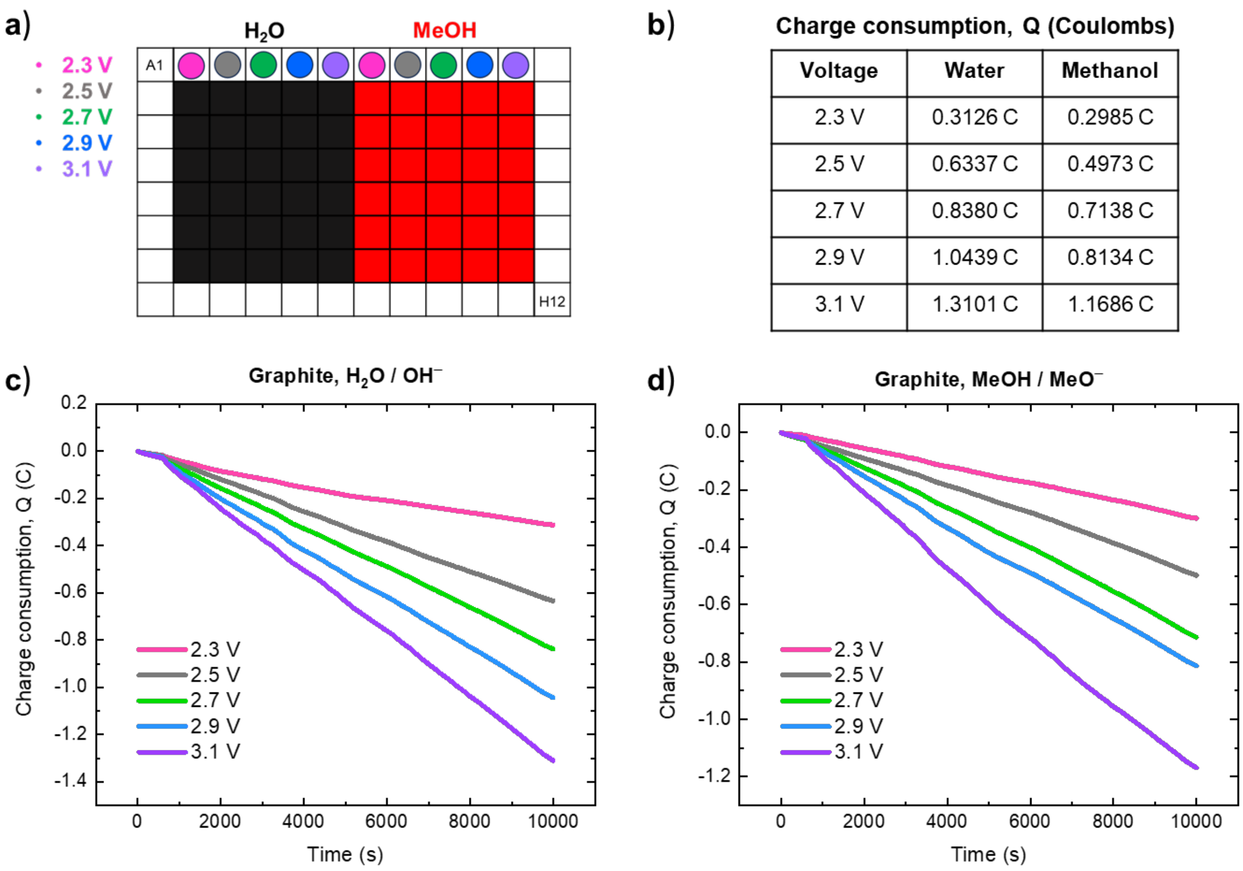

Charge consumption, Q (Coulombs)

Voltage	Water	Methanol
2.3 V	0.3126 C	0.2985 C
2.5 V	0.6337 C	0.4973 C
2.7 V	0.8380 C	0.7138 C
2.9 V	1.0439 C	0.8134 C
3.1 V	1.3101 C	1.1686 C

Figure S3. Arrayed controlled potential electrolysis (CPE) for approximately 2.75 hours at a graphite working electrode surface. Arrays contain a 2-electrode configuration: graphite plate working electrode and platinized titanium (Pt-Ti) wire quasi-reference counter electrode. Electrolyses are conducted in 300 µL of 0.1 M KPF_6 supporting electrode in acetonitrile, with 2 mM KI, 20 mM benzophenone imine, and 200 mM of HA / A^-.

Arrays consist of (a) water (*black*) and methanol (*red*) as acids and conjugated bases, as well as varying applied potentials. (b) Charge consumption over the course of 2.75 hours of electrolysis. Charge *vs*. time plots in the presence of (c) 200 mM water, and (d) 200 mM methanol. Each charge *vs*. time plot and charge consumption value represents the average measurement across one corresponding set of eight wells.

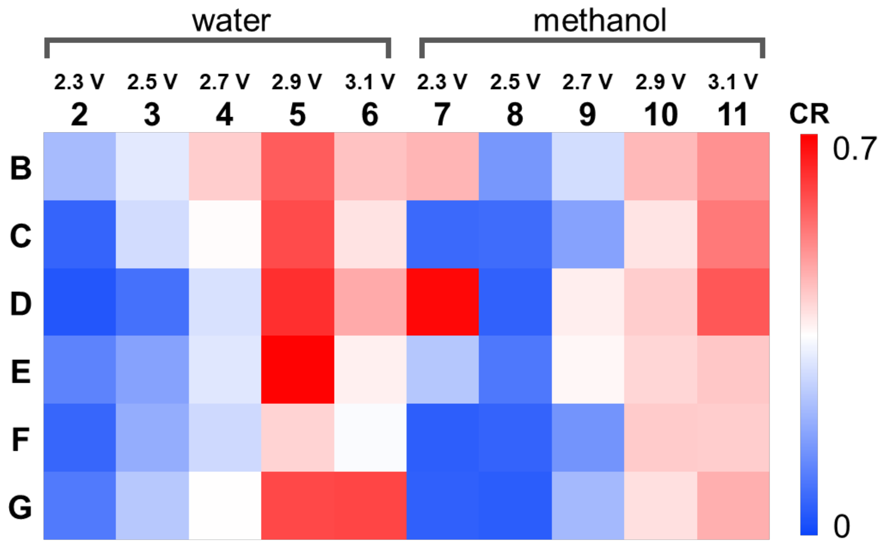

Figure S4. Heatmap for electrosynthesis of benzophenone azine with graphite using different potentials and proton source, analyzed by chip-based nESI-MS. Heatmap was generated using conversion ratio (CR) calculated with MS ion intensities.

	1	2	3	4	5	6	7	8	9	10	11	12
Average	0.0114	0.0190	0.0829	0.1680	0.137	-	0.0123	0.0231	0.0744	0.191	0.161	-
Standard Deviation	0.00689	0.0116	0.0400	0.0451	0.0310	-	0.00199	0.0130	0.0316	0.0340	0.0823	-

Figure S5. Distribution of conversion ratios across the 96-well array, after controlled potential electrolysis at a glassy carbon plate. Channels 6 and 12 have been omitted, as they contain only supporting electrolyte and generate no benzophenone azine product. In this case, Cell H11 was omitted as outlier after short-circuiting during electrosynthesis.

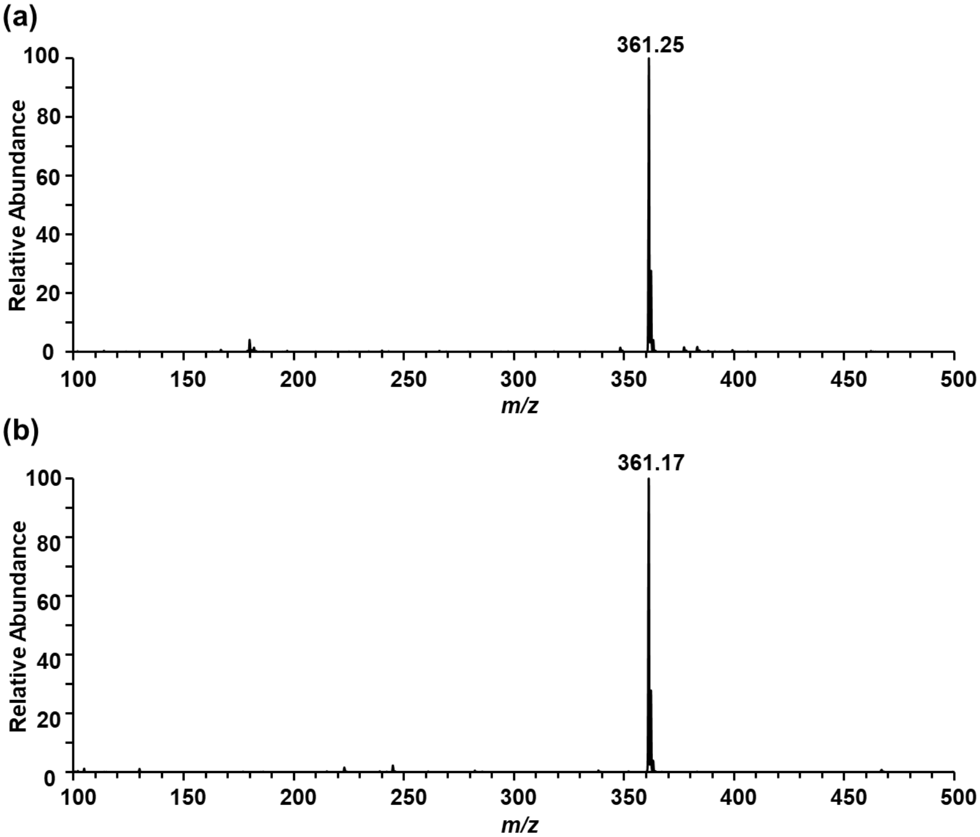

Figure S6. Comparison of (a) electrosynthesized benzophenone azine at *m/z* 361 (100 μM assuming 100% conversion) to (b) 100 μM benzophenone azine commercial standard.

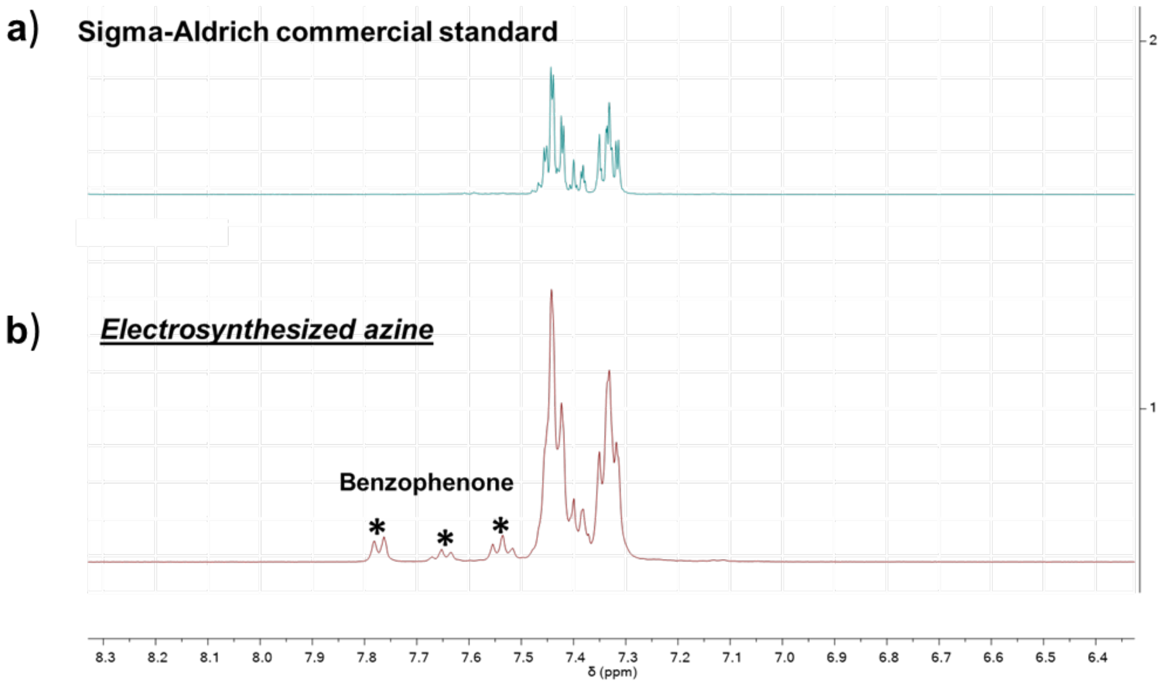

Figure S7. ^{1}H-NMR in CD_3CN of (a) as-purchased benzophenone azine (*teal*), and (b) electrosynthesized benzophenone azine (*dark red*). Commercial benzophenone imine starting material contains 3% benzophenone, which is inert during electrolysis. 50 mM mesitylene as internal standard (δ = 6.7970, 6.7955 ppm).

References

(1) Gerroll, B. H. R.; Kulesa, K. M.; Ault, C. A.; Baker, L. A. Legion: An instrument for high-throughput electrochemistry. *ACS Meas. Sci. Au* **2023**, *3*, 371-379.
